# Adrenocortical steroid response to ACTH in different phenotypes of non-obese polycystic ovary syndrome

**DOI:** 10.1186/1757-2215-5-42

**Published:** 2012-12-07

**Authors:** Nese Cinar, Ayla Harmanci, Duygu Yazgan Aksoy, Kadriye Aydin, Bulent Okan Yildiz

**Affiliations:** 1 Endocrinology and Metabolism Unit, Department of Internal Medicine, Hacettepe University School of Medicine Hacettepe, Ankara, 06100, Turkey

**Keywords:** Adrenal androgen, PCOS, ACTH, DHEAS

## Abstract

**Background:**

Adrenal androgen excess is frequently observed in PCOS. The aim of the study was to determine whether adrenal gland function varies among PCOS phenotypes, women with hyperandrogenism (H) only and healthy women.

**Methods:**

The study included 119 non-obese patients with PCOS (age: 22.2 ± 4.1y, BMI:22.5 ± 3.1 kg/m^2^), 24 women with H only and 39 age and BMI- matched controls. Among women with PCOS, 50 had H, oligo-anovulation (O), and polycystic ovaries (P) (PHO), 32 had O and H (OH), 23 had P and H (PH), and 14 had P and O (PO). Total testosterone (T), SHBG and DHEAS levels at basal and serum 17-hydroxprogesterone (17-OHP), androstenedione (A4), DHEA and cortisol levels after ACTH stimulation were measured.

**Results:**

T, FAI and DHEAS, and basal and AUC values for 17-OHP and A4 were significantly and similarly higher in PCOS and H groups than controls (p < 0.05 for all) whereas three groups did not differ for basal or AUC values of DHEA and cortisol. Three hyperandrogenic subphenotypes (PHO, OH, and PH) compared to non-hyperandrogenic subphenotype (PO) had significantly and similarly higher T, FAI, DHEAS and AUC values for 17-OHP, A4 and DHEA (p < 0.05). All subphenotypes had similar basal and AUC values for cortisol.

**Conclusion:**

PCOS patients and women with H only have similar and higher basal and stimulated adrenal androgen levels than controls. All three hyperandrogenic subphenotypes of PCOS exhibit similar and higher basal and stimulated adrenal androgen secretion patterns compared to non-hyperandrogenic subphenotype.

## Introduction

Polycystic ovary syndrome (PCOS) is the most common endocrine disorder of reproductive-aged women with an estimated prevalence of 6-7% [1]. PCOS is characterized by androgen excess, oligo-anovulation (O) and polycystic ovaries (P). Since it’s a heterogeneous disorder, several criteria have been proposed for its diagnosis [2,3]. According to 1990 National Institutes of Health (NIH) criteria, the presence of both oligo-and/or anovulation and clinical (hirsutism) and /or biochemical signs of hyperandrogenism (H) are needed, regardless of the presence of P on ultrasound [2]. Due to the lack of agreement on standardized criteria to make the diagnosis of PCOS, an international consensus workshop in Rotterdam, sponsored by The American Society for Reproductive Medicine (ASRM) and European Society for Human Reproduction and Embryology (ESHRE) expanded the diagnostic criteria for PCOS with the addition of the ultrasound assesment of ovarian morphology [3]. According to these new criteria, PCOS can be defined when at least two of the three features (O,H and P) are present. Using these Rotterdam criteria, four phenotypes of PCOS are identified (i.e.) PHO (phenotype1), OH (phenotype 2), PH (phenotype 3) and PO (phenotype 4).

While the ovaries are the main source of androgen excess in PCOS, excess adrenal androgen (AA) levels and adrenocortical dysfunction have been reported in many PCOS patients [4-7]. Elevated serum levels of dehydroepiandrosterone sulfate (DHEAS) and 11β-hydroxyandrostenedione (11-OHA4) is found in 20-50% of patients with PCOS [4,6]. PCOS women show a generalized hypersecretion of adrenocortical products, basally and in response to ACTH including pregnenolone, 17-hydroxypregnenolone, dehydroepiandrosterone (DHEA), androstenedione (A4) and possibly cortisol (F) [8].

Peripubertal AA excess is associated with the development of PCOS-like symptomatology in patients with 21-hydroxylase deficient classic and non-classic adrenal hyperplasia [9,10]. Patients with PCOS have a greater incidence of exaggerated AA secretion during the peripubertal period [11] and patients with premature adrenarche are at higher risk for the development of PCOS [12]. Moreover, women with PCOS exhibit enhanced adrenal androgen production over long time [13,14] and increased DHEAS levels were reported in sisters [15] of PCOS patients suggesting that inheritance plays a significant role in AA secretion of PCOS.

Previous data suggest that adrenocortical excess in PCOS might result in different phenotypic features (e.g. acne) [16]. Recently, differences in metabolic characteristics and basal AA levels of the subphenotypes of PCOS have been reported indicating phenotype PO as the mildest expression of the PCOS spectrum [17,18]. We now hypothesize that AA excess will be less pronounced in the phenotype PO of PCOS and will be more associated with hyperandrogenic phenotypes. To test our hypothesis, we determine the differences in AA production in response to ACTH stimulation among different phenotypes of PCOS according to Rotterdam criteria, women with H only and healthy women.

## Materials and methods

### Subjects

A total of 143 consecutive non-obese patients (BMI < 30 kg/m^2^), who attended to the Outpatient Clinic of Endocrinology and Metabolism of Hacettepe University between January 1 2006 and December 31 2010 were prospectively enrolled in the study. Among the patients, 50 had H, O and P (PHO), 32 had H and O without P (OH), 23 had H and P with normal menses (PH), 14 had O and P (PO) and 24 women with H only. All patients fulfilled the diagnostic criteria for PCOS according to revised 2003 Rotterdam criteria [3]. H was defined as having hirsutism (modified Ferriman-Gallwey score ≥ 6) and/or a total testosterone (T) and/or free androgen index (FAI) level above the upper 95th percentile of 110 healthy non-hirsute eumenorrheic women. Specifically the upper normal limits were as follows: total T, 60 ng/dl (2.08 nmol/liter) and FAI, 4.94. Ovulatory dysfunction was defined as menstrual cycles ≥35 or ≤23 days. In patients with hirsutism or P appearance who had apparently regular menstrual bleeding, luteal phase (Days 21–24) progesterone levels were determined and the threshold for the presence of ovulation was taken as 4 ng/ml (12.7 nmol/liter). P was defined as the presence of ≥12 follicles in an ovary measuring 2–9 mm in diameter and/or increased ovarian volume (>10 mL) at least in a single ovary [3]. Women with a secondary endocrine disorder, such as hyperprolactinaemia, non-classical form of congenital adrenal hyperplasia, thyroid function disorder and androgen secreting tumors were excluded as suggested [3]. Thirty-nine age- and BMI- matched healthy women without features of H, O or P formed the control group. None of the subjects were taking any hormonal medication including oral contraceptive pills for at least 3 months before the study.

The study was approved by the Local Ethics Committee of the Hacettepe University School of Medicine and informed consent was obtained from all subjects.

### Study protocol

All participants were evaluated by means of a standardized form that includes a medical history and physical examination. Weight, height, waist and hip circumferences (waist: midway between the lower rib margin and the iliac crest, hip: widest circumference over the great trochanters) were measured. The body mass index [BMI; weight (kilograms)/ height (meters) ^2^] and waist to hip ratio (WHR) were calculated. Hirsutism was evaluated by modified Ferriman-Gallwey scoring (mFG) system.

All subjects were studied during the follicular phase of the menstrual cycle (2–5 days after onset of last spontaneous or progestin-induced menstrual bleeding). Basal blood samples were obtained for hormonal and metabolic parameters. Hormonal and metabolic evaluation included T, A4, DHEAS and sex hormone-binding globulin (SHBG), fasting plasma glucose (FPG) and insulin. Free androgen index (FAI = [testosterone (nmol/L)/SHBG(nmol/L)]x100) and homeostatic model assesment of insulin resistance (HOMA-IR = [fasting insulin (μU/ml)x fasting plasma glucose (FPG) (mmol/L)]/22.5) were calculated as previously described [19].

All participants underwent short ACTH stimulation test. 0.25 mg ACTH-(1-24) (Synacthen 0.25 mg/1 ml; Defiante Farmaceutica, S.A., Portugal) was administered IV over 60 sec. and blood samples were drawn for serum F, 17-OHP, A4 and DHEA levels 1 min. before and 30 and 60 min. after ACTH administration. Areas under the curve (AUC), and delta max values for 17-OHP, A4, DHEA and F responses were calculated.

### Assays

Blood samples were taken through venepuncture and centrifuged within 2 h after withdrawal. Serum was stored at −80°C until time of studies. Testosterone and insulin concentrations were measured by chemiluminiscent immunoassay kits (Roche Diagnostics GmbH, Mannheim, Germany). SHBG was measured by using immunoradiometric assay (ZenTech, Angleur, Belgium). Plasma glucose concentration was determined by the glucose oxidase method (Olympus AU 2700, Beckmann,Coulter, Inc., MA, USA). The concentration of DHEAS and A4 were determined by chemiluminiscent immunoassay (Immulite® 2000, Los Angeles, USA). Serum DHEA level was measured using a solid phase enzyme-linked immunosorbent assay (DRG Instruments, Marburg, Germany). 17-OHP was measured by radioimmunoassay kit from Immunotech.

### Statistical analysis

Differences in basal characteristics and laboratory data among the groups were analyzed by one-way analysis of variance (ANOVA) and post hoc Tukey test for dual-wise comparisons for normally distributed parameters, and Kruskal-Wallis one-way ANOVA and Mann–Whitney test for dual-wise comparisons for parameters with skewed distribution. By using the trapezoidal method, 17-OHP, A4, DHEA and F responses to ACTH stimulation were expressed as AUC. Pearson/Spearman correlations were used to examine the relationship between DHEAS levels and fasting insulin and HOMA-IR values. Statistical Package for Social Sciences, version 15.0 (SPSS Inc., Chicago) was used for analyses. Values were described as mean ± SD and *P* < 0.05 was considered statistically significant.

## Results

The basal characteristics of the PCOS, H only and control groups are shown in Table 1. No significant difference in the basal characteristics was observed between PCOS and H only group (Table 1, NS for all). The mean levels of T, FAI and DHEAS were significantly higher in PCOS and H only group than controls (Table 1, P < 0.001 for all). There was no significant difference in FPG, fasting insulin and HOMA-IR values among the groups (Table 1, NS for all). Basal, stimulated and AUC values for 17-OHP and A4 were significantly and similarly higher in PCOS and H only groups than controls (Table 2, P < 0.01 for all) whereas three groups did not differ for basal, stimulated or AUC values of DHEA and F (Table 2, NS for all). Also, delta max AA responses to ACTH were evaluated and PCOS and H only group had higher values for 17OHP and A4 than controls (P < 0.05 for all, data not shown).

**Table 1:** Baseline clinical and hormonal characteristics of the women with PCOS, women with H only and control groups

**Parameters**	**PCOS**	**H**	**Control**	**P**
	**(n = 119)**	**(n = 24)**	**(n = 39)**	**(overall)**
**Age** (year)	22.2 ± 4.1	23.8 ± 4.9	24.4 ± 2.9	NS
**BMI** (kg/m^2^)	22.5 ± 3.1	22.1 ± 2.3	21.2 ± 2.08	NS
**WHR**	0.76 ± 0.06	0.77 ± 0.04	0.75 ± 0.06	NS
**mFG**	8.7 ± 5.3ª	10.0 ± 3.4ª	0.6 ± 0.9	<0.001
**Testosterone** (ng/dl)	70.4 ± 34.5ª	70.3 ± 30.7ª	34.9 ± 11.6	<0.001
**SHBG** (nmol/l)	39.6 ± 20.7ª	32.8 ± 16.7ª	56.5 ± 29.1	<0.001
**FAI**	8.8 ± 7.6ª	8.5 ± 4.5ª	2.7 ± 1.6	<0.001
**DHEAS** (μg/dl)	278 ±134^b^	314 ± 115ª	204 ± 74	<0.001
**FPG** (mg/dl)	82.4 ± 11.8	81.2 ± 10.7	83.7 ± 11.2	NS
**Fasting insulin** (μIU/ml)	11.1 ± 5.9	9.2 ± 3.5	9.4 ± 3.1	NS
**HOMA-IR**	2.3 ± 1.3	1.8 ± 0.7	1.9 ± 0.7	NS

Results are expressed as mean ± SD. *NS* not significant, *BMI* body mass index, *WHR* waist-to-hip ratio, *mFG* modified Ferriman-Gallwey score, *SHBG* sex hormone-binding globulin, *FAI* free androgen index, *DHEAS* dehydroepiandrosterone sulfate, *FPG* fasting plasma glucose, *HOMA*-*IR* homeostatic model assesment of insulin resistance.

^a^ P <0.001 vs. Control.

^b^ P <0.01 vs. Control.

**Table 2:** ACTH stimulated hormone levels and their AUC values in patients with PCOS, women with H only and control groups

**Parameters**	**PCOS**	**H**	**Control**	**P**
	**(n = 119)**	**(n = 24)**	**(n = 39)**	**(overall)**
**DHEA**_**0**_	16.7 ± 7.9	20.7 ± 8.9	14.5 ± 5.9	NS
**DHEA**_**30**_	23.8 ± 6.8	25.2 ± 6.2	21.5 ± 6.3	NS
**DHEA**_**60**_	25.4 ± 6.3	26.3 ± 6.0	24.6 ± 6.3	NS
**17-OHP**_**0**_ (ng/ml)	1.5 ± 0.9^b^	1.6 ± 0.7^b^	1.1 ± 0.7	<0.01
**17-OHP**_**30**_ (ng/ml)	3.5 ± 1.5^b^	3.4 ± 0.9^c^	2.8 ± 1.2	<0.01
**17-OHP**_**60**_ (ng/ml)	4.1 ± 1.7^b^	3.9 ± 0.9^b^	3.2 ± 1.1	<0.01
**A4**_**0**_ (ng/ml)	3.1 ± 1.5^a^	3.2 ± 1.2^b^	2.2 ± 0.9	<0.001
**A4**_**30**_ (ng/ml)	4.0 ± 1.8^a^	4.0 ± 1.2^b^	2.8 ± 0.9	<0.001
**A4**_**60**_ (ng/ml)	4.3 ± 1.7^a^	4.1 ± 1.2^b^	3.1 ± 0.9	<0.001
**F**_**0**_ (μg/dl)	13.9 ± 5.8	15.9 ± 7.6	14.0 ± 5.4	NS
**F**_**30**_ (μg/dl)	26.5 ± 5.0	26.2 ± 5.4	25.9 ± 4.5	NS
**F**_**60**_ (μg/dl)	30.9 ± 6.1	30.8 ± 6.4	30.6 ± 5.0	NS
**AUC**_DHEA_ (ng/ml × 60 min)	1338 ± 375	1459 ± 375	1227 ± 351	NS
**AUC**_17-OHP_ (ng/ml × 60 min)	190 ± 80^b^	183 ± 44^b^	148 ± 60	<0.01
**AUC**_A4_ (ng/ml × 60 min)	232 ± 99^a^	229 ± 69^c^	165 ± 53	<0.001
**AUC**_F_ (μg/dl × 60 min)	1471 ± 303	1487 ± 361	1446 ± 258	NS

Results are expressed as mean ± SD. *NS* not significant, *AUC* area under the curve, *DHEA* dehydroepiandrosterone, *17-OHP* 17-hydroxyprogesterone, *A4* androstenedione, *F* cortisol.

^a^ P <0.001 vs. Control.

^b^ P <0.01 vs. Control.

^c^ P <0.05 vs. Control.

Table 3 shows the comparisons of clinical and hormonal characteristics among different phenotypes of PCOS. All the phenotypes had similar mean age, BMI and WHR values (Table 3, NS for all). Within the PCOS group, 3 hyperandrogenic subphenotypes (PHO, OH and PH) compared to non-hyperandrogenic subphenotype (PO) had significantly and similarly higher T, FAI, DHEAS (Table 3, P < 0.001 for all). While there was no significant difference in FPG levels among the groups (Table 3, NS), a significant difference in fasting insulin levels and HOMA-IR values between PHO, OH and PH groups was observed (Table 3, P < 0.01 for all). Stimulated and AUC values for 17-OHP and A4 were significantly higher in 3 hyperandrogenic subphenotypes (PHO, OH and PH) than non-hyperandrogenic subphenotype (PO) (Table 4, P < 0.05 for all). All subphenotypes had similar basal, stimulated and AUC values for F (Table 4, NS for all). Basal, stimulated and AUC values for DHEA in OH and PH groups were significantly higher than those in PO group (Table 4, P < 0.05 for all).

**Table 3:** Comparisons of clinical and hormonal characteristics among different phenotypes of PCOS

**Parameters**	**PHO**	**OH**	**PH**	**PO**	***P***
	**(n = 50)**	**(n = 32)**	**(n = 23)**	**(n = 14)**	**(overall)**
**Age** (year)	22.4 ± 4.5	22.4 ± 4.3	22.3 ± 3.3	20.8 ± 3.4	NS
**BMI** (kg/m^2^)	22.6 ± 3.1	22.7 ± 3.2	22.1 ± 2.9	22.5 ± 4.04	NS
**WHR**	0.78 ± 0.05	0.77 ± 0.05	0.75 ± 0.06	0.77 ± 0.07	NS
**mFG**	8.2 ± 5.0^a,*^	10.9 ± 5.1^a^	10.6 ± 3.6^a^	1.4 ± 1.7	<0.001
**Testosterone** (ng/dl)	74.8 ± 37.6^a^	77.9 ± 30.8^a^	71.7 ± 29.1^a^	35.9 ± 15.7	<0.001
**SHBG** (nmol/l)	39.9 ± 23.5	36.9 ± 17.8	38.6 ± 20.7	46.0 ± 15.7	NS
**FAI**	9.9 ± 8.4^a^	9.6 ± 8.0^a^	8.8 ± 5.8^b^	2.8 ± 1.4	<0.01
**DHEAS** (μg/dl)	277 ± 127 ^b^	312 ±140^b^	294 ± 136^b^	174.9 ± 95.8	<0.01
**FPG** (mg/dl)	82.0 ± 12.6	81.4 ± 11.5	83.1 ± 9.3	84.9 ± 14.4	NS
**Fasting insulin** (μIU/ml)	12.3 ± 7.5^c^	11.2 ± 3.7^c^	8.2 ± 4.1	10.2 ± 4.9	<0.01
**HOMA-IR**	2.6 ± 1.6^c^	2.3 ± 0.8^c^	1.7 ± 0.9	2.3 ± 1.2	<0.05

Results are expressed as mean ± SD. *NS* not significant, *PHO* polycystic ovaries, oligo-anovulation and hyperandrogenism, *OH* oligo-anovulation and hyperandrogenism, *PH* polycystic ovaries and hyperandrogenism, *PO* polycystic ovaries and oligo-anovulation, *BMI* body mass index, *WHR* waist-to-hip ratio, *mFG* modified Ferriman-Gallwey score, *SHBG* sex hormone-binding globulin, *FAI* free androgen index, *DHEAS* dehydroepiandrosterone sulfate, *FPG* fasting plasma glucose, *HOMA-IR* homeostatic model assesment of insulin resistance.

^a^ P <0.001 vs. PO.

^b^ P <0.01 vs. PO.

^c^ P <0.01 vs. PH.

*P =0.05 vs. OH.

**Table 4:** ACTH stimulated hormone levels and their AUC values among different phenotypes of PCOS

**Parameters**	**PHO**	**OH**	**PH**	**PO**	***P***
	**(n = 50)**	**(n = 32)**	**(n = 23)**	**(n = 14)**	**(overall)**
**DHEA**_**0**_	15.9 ± 7.9	19.3 ± 7.7^a^	17.2 ± 7.3^b^	12.2 ± 7.5	<0.05
**DHEA**_**30**_	23.7 ± 6.7^b^	24.9 ± 6.7^a^	25.5 ± 5.5^a^	18.2 ± 6.4	<0.05
**DHEA**_**60**_	24.7 ± 6.3*	27.2 ± 5.9^b^	26.3 ± 5.2	21.8 ± 8.1	<0.05
**17-OHP**_**0**_ (ng/ml)	1.6 ± 1.1	1.4 ± 0.7	1.6 ± 1.3	0.9 ± 0.3	0.078
**17-OHP**_**30**_ (ng/ml)	3.7 ± 1.7^b^	3.7 ± 1.2^a^	3.6 ± 1.5^b^	2.6 ± 1.1	<0.05
**17-OHP**_**60**_ (ng/ml)	4.3 ± 1.9	4.2 ± 1.5	4.3 ± 1.8	3.1 ± 1.1	0.072
**A4**_**0**_ (ng/ml)	3.4 ± 1.9	3.2 ± 1.2	2.8 ± 0.8	2.2 ± 1.0	NS
**A4**_**30**_ (ng/ml)	4.3 ± 2.0^a^	4.3 ± 1.8^b^	4.0 ± 1.3^b^	2.9 ± 0.9	<0.05
**A4**_**60**_ (ng/ml)	4.7 ± 1.9^a^	4.3 ± 1.7^b^	4.4 ± 1.4^a^	3.1 ± 1.0	<0.01
**F**_**0**_ (μg/dl)	14.4 ± 6.3	14.6 ± 6.0	13.9 ± 5.6	11.6 ± 4.4	NS
**F**_**30**_ (μg/dl)	26.2 ± 4.6	28.6 ± 5.1	25.9 ± 6.1	25.2 ± 4.6	NS
**F**_**60**_ (μg/dl)	30.4 ± 6.7	32.3 ± 5.7	31.5 ± 5.9	29.5 ± 5.5	NS
**AUC**_DHEA_ (ng/ml × 60 min)	1314 ± 390	1437 ± 348^a^	1415 ± 297^a^	1057 ± 397	<0.05
**AUC**_17-OHP_ (ng/ml × 60 min)	198 ± 90^a^	195 ± 62^a^	194 ± 85^b^	140 ± 51	<0.05
**AUC**_A4_ (ng/ml × 60 min)	254 ± 117^a^	240 ± 93^b^	230 ± 69^a^	164 ± 55	<0.05
**AUC**_F_ (μg/dl × 60 min)	1466 ± 312	1563 ± 298	1456 ± 331	1372 ± 253	NS

Results are expressed as mean ± SD. *NS* not significant, *AUC* area under the curve, *PHO* polycystic ovaries, oligo-anovulation and hyperandrogenism, *OH* oligo-anovulation and hyperandrogenism, *PH* polycystic ovaries and hyperandrogenism, *PO* polycystic ovaries and oligo-anovulation, *DHEA*, dehydroepiandrosterone, *17-OHP* 17-hydroxyprogesterone, *A4* androstenedione, *F* cortisol.

^a^ P <0.01 vs. PO.

^b^ P <0.05 vs. PO.

*P <0.05 vs. OH.

To evaluate the prevalence of AA excess in the study groups, DHEAS levels were log transformed to satisfy the assumption of the normal distribution and the upper 95% normative value for log DHEAS for control group was calculated. 95th percentile log DHEAS value in the control group was found as 2.54. The prevalence rates of supranormal DHEAS levels were 27.6% in PCOS, 33.3% in H only group and 2.6% in the control group respectively. PCOS and H only groups had significantly higher rates of AA excess than controls (27.6%, 33.3% and 2.6% respectively; P < 0.05) while the two groups did not differ in comparison. Among the PCOS phenotypes, PHO and OH group had non-significantly higher rates of AA excess than non-hyperandrogenic subphenotype (PO) (PHO 31.3% vs PO 7.1% P = 0.09; OH 34.4% vs PO 7.1% P = 0.073) whereas no significant difference was observed between PH and PO group (22.7% vs 7.1% respectively, NS).

DHEAS levels did not show any correlation with fasting insulin or HOMA-IR values in PCOS, H only or control groups or in subphenotype groups of PCOS (NS, data not shown).

## Discussion

In this study, we report adrenocortical steroid response to ACTH among patients with different phenotypes of PCOS according to Rotterdam criteria, women with H only and healthy women. Our data indicate that non-obese patients with PCOS and women with H only have similar adrenal responses that differ from age- and BMI-matched healthy women with higher basal DHEAS, and higher 17-OHP and A4 levels both basally and in response to ACTH. Even though hyperandrogenism was defined by the presence of hirsutism and/or increased FAI in this study, all three hyperandrogenic subphenotypes exhibited similar AA secretion patterns with higher DHEAS, and higher 17-OHP and A4 responses to ACTH stimulation compared to non-hyperandrogenic subphenotype. Similar basal DHEA levels in PCOS, H only and control women despite higher DHEAS values in PCOS and H only groups compared to healthy women might suggest differences in the expression of DHEA-sulfotransferase activity.

Adrenarche is the maturation of the zona reticularis of the adrenal gland with increase in AAs in early puberty. Premature and/or exaggerated adrenarche is reported to be associated with the development of PCOS in a number of studies [20,21]. Regarding the role of AAs in the pathophysiology of PCOS, adrenal function during childhood and pubertal development in daughters of women with PCOS was studied [22]. In this study, increased DHEAS serum concentrations and biochemical evidence of an exacerbated adrenarche was observed in daughters of women with PCOS suggesting these features as an early step in the development of PCOS [22]. Moreover, adrenocortical excess in PCOS might result in different phenotypic features (e.g.acne) [16]. It is also important to note that the alterations in androgen levels with aging in PCOS might result in changes in phenotypic expression of the syndrome (i.e. decrease in prevalence of hyperandrogenic phenotypes) [23]. Our data suggest that hyperandrogenic subphenotypes of PCOS have increased prevalence of AA excess in the form of circulating DHEAS levels and higher AA response to ACTH stimulation compared to non-hyperandrogenic subphenotype. Taken together, it might be postulated that the subjects who exposed to premature and/or exaggerated adrenarche during pubertal development might present with hyperandrogenic phenotypes of PCOS later in life and AAs might play role in the clinical presentation and phenotypic expression of PCOS.

Enhanced secretory response of A4, 17-OHP and DHEA after ACTH stimulation in women with PCOS defined by NIH criteria was shown in the previous studies [24-26]. Conflicting results are also available even in studies using the same NIH criteria [27,28]. For example, Erel et al. showed that AA response to ACTH was similar between women with PCOS and controls [27], whereas Kamel et al. reported that PCOS patients (n = 29) had higher basal and stimulated DHEAS, 17-OHP and stimulated F levels than idiopathic hirsutism (IH) (n = 21) and control (n = 20) subjects [28]. Evaluating potential differences of AA excess among various PCOS subphenotypes we now provide data supporting the hypothesis that AA excess both basally and in reponse to ACTH is more associated with hyperandrogenic PCOS and is not a prominent feature of the phenotype formed by ovulatory dysfunction and polycystic ovaries. Our data are in line wih previous work in that basal and stimulated cortisol levels in PCOS are similar with healthy women [29]. Furthermore, cortisol responses to ACTH appear to be similar in subphenotypes of PCOS as well.

Prevalence of AA excess defined by supranormal DHEAS levels in PCOS is between 20-30% . Carmina et al. reported similar prevalence of AA excess determined by elevations of DHEAS in idiopathic hyperandrogenism (IHA), classic PCOS and ovulatory PCOS (PH) (48.3%, 36.7% and 29.1% respectively, NS) [30]. We have found similar rates of AA excess in PCOS, H only patients and among hyperandrogenic phenotypes of PCOS (PHO, OH, PH).

IHA, a diagnosis exclusion, is the second most common androgen disorder. Adrenal hyperandrogenism is common in these patients. Almost 50% of the patients with IHA were reported to have elevated circulating DHEAS levels [30]. An isolated increase of DHEAS levels was found in 7% of the patients and ovarian source of the androgens was found in 90% of the patients associated with exaggerated AA production in about half of them [30,31]. Atmaca et al. showed that peak and AUC responses of 11-deoxy-cortisol, DHEAS and A4 to ACTH were significantly higher in IHA patients than controls [32]. These data are in line with our finding of similar adrenal hyperfunction in PCOS and H only groups that were higher than controls.

Comparison of endocrine and metabolic characteristics of the different phenotypes of PCOS were studied in several studies [17,18,33,34] . Panidis et al. reported that circulating androgens were higher in PCOS patients with hyperandrogenic subphenotypes (PHO, OH and PH) compared with those with non-hyperandrogenic subphenotype (PO) [17]. Kauffman et al. also showed that testosterone and DHEAS levels were highest in PHO and OH phenotype and the androgen levels were indistinguishable in PO than controls [33]. These results suggest that androgen production is the most prominent endocrine and metabolic factor differentiating phenotypic expressions of PCOS and phenotype PO is the mildest expression of the PCOS spectrum. In accordance with these data, we have found that AA excess both basally and in response to ACTH was less pronounced in the phenotype PO of PCOS and more associated with hyperandrogenic phenotypes.

Conflicting data are available regarding potential interaction between glucose/insulin axis and adrenocortical dysfunction in PCOS. Hyperinsulinemia in PCOS stimulates androgen secretion by ovarian theca cells and increases the hormonally active free androgen fraction by reducing the hepatic production of SHBG [35]. Insulin sensitizing agents such as metformin causes a decrease in serum androgen levels with improvement in insulin sensivity parameters [36]. In a study including 1212 women with PCOS and 254 healthy women, phenotype PHO was found to be associated with more IR than other phenotypes [17]. Obese patients with OH and PO phenotype were also characterized with IR whereas phenotype PH was not associated with IR. No significant association was reported between DHEAS levels and fasting insulin in some studies [37] whereas high DHEAS levels were reported to be negatively correlated to insulin resistance in others [16,38,39]. Moreover, it was demonstrated that hyperinsulinemia potentiates ACTH-stimulated androgen production in women with PCOS [40]. Regarding the association between insulin resistance and hyperandrogenism, studies with insulin sensitizers such as thiazolidinediones or metformin showed that significant reductions were observed in the secretion of A4 and 17-OHP in response to ACTH stimulation [41,42]. On the other hand, adrenocortical biosynthesis, basally and in response to ACTH, are thought to be associated with glucose-mediated glucose disposal rather than the degree of hyperinsulinemia or insulin-mediated glucose disposal [43]. In our study, we have failed to show any difference in insulin resistance parameters between PCOS patients and controls, and a relationship between DHEAS levels and fasting insulin or HOMA-IR values. Normal BMI and relatively young age of the participants might in part explain our finding of a non-significant difference in fasting insulin and HOMA-IR values between patients and controls. Alternatively, we might have failed to detect a difference due to insensitivity of the fasting measurements we have used. Obesity may affect adrenocortical function by decreasing insulin sensitivity and inducing the secretion of adipocytokines. Available literature suggests that hypersecretion of F and possibly A4 and DHEA are observed in obese healthy women [44] although conflicting results are also reported [45]. Our study design precludes us from any potential confounding effect of obesity since only lean women participated in the study.

A limitation of our study is the relatively small sample size of the groups with each phenotype. Secondly, we were not able to assess potential ovarian contribution to androgen synthesis since we did not use a GnRH analogue. Thirdly, our results could not be extrapolated to obese women with PCOS or PCOS patients of different races.

In conclusion, our data suggest that in PCOS patients and women with H only, basal and ACTH-stimulated AA levels are similar and higher than those in healthy women. All three hyperandrogenic subphenotypes of PCOS exhibit similar and higher basal and ACTH-stimulated AA secretion patterns compared to non-hyperandrogenic subphenotype. AA excess appears to be more associated with hyperandrogenism and is less pronounced in women with ovulatory dysfunction and polycystic ovaries. Similar AA production in women with H only and hyperandrogenic subphenotypes of PCOS suggest that this group might be a spectrum of PCOS and might develop the syndrome at a later time. Understanding the regulation of adrenal steroidogenesis in subphenotypes of PCOS might provide novel mechanistic insights into the pathophysiology and developmental context of the syndrome.

## Competing interests

The authors declare that they have no competing interests.

## Authors’ contribution

NC: contributed to acquisition, analysis and interpretation of the data, and drafting the article. AH: contributed to acquisition of the data, and critical revision of the paper. DYA: contributed to acquisition of the data, and critical revision of the paper. KA: contributed to acquisition of the data, and critical revision of the paper. BOY: contributed to conception and design, acquisition, analysis and interpretation of the data, and critical revision of the paper. All authors read and approved the final manuscript.
